# The Ferroxidase Hephaestin in Lung Cancer: Pathological Significance and Prognostic Value

**DOI:** 10.3389/fonc.2021.638856

**Published:** 2021-05-19

**Authors:** Paola Zacchi, Beatrice Belmonte, Alessandro Mangogna, Gaia Morello, Letizia Scola, Anna Martorana, Violetta Borelli

**Affiliations:** ^1^ Department of Life Sciences, University of Trieste, Trieste, Italy; ^2^ Tumor Immunology Unit, Department of Health Sciences, University of Palermo, Palermo, Italy; ^3^ Institute for Maternal and Child Health, IRCCS Burlo Garofolo, Trieste, Italy; ^4^ Clinical Pathology, Department of Biomedicine, Neuroscience and Advanced Diagnostics (Bi.N.D.), University of Palermo, Palermo, Italy

**Keywords:** lung cancer, hephaestin, iron, immunohistochemistry, bioinformatics

## Abstract

Hephaestin (HEPH) belongs to a group of exocytoplasmic ferroxidases which contribute to cellular iron homeostasis by favouring its export. Down-regulation of HEPH expression, possibly by stimulating cell proliferation due to an increase in iron availability, has shown to correlate with poor survival in breast cancer. The lung is particularly sensitive to iron-induced oxidative stress, given the high oxygen tension present, however, HEPH distribution in lung cancer and its influence on prognosis have not been investigated yet. In this study we explored the prognostic value of HEPH and its expression pattern in the most prevalent histotypes of lung cancers, namely lung adenocarcinoma and lung squamous cell carcinoma. *In silico* analyses, based on UALCAN, Gene Expression Profiling Interactive Analysis (GEPIA) and Kaplan–Meier plotter bioinformatics, revealed a significant correlation between higher levels of HEPH expression and favorable prognosis, in both cancer histotypes. Moreover, TIMER web platform showed a statistically significant association between HEPH expression and cell elements belonging to the tumor microenvironment identified as endothelial cells and a subpopulation of cancer-associated fibroblasts, further confirmed by double immunohistochemical labeling with cell type specific markers. Taken together, these data shed a light on the complex mechanisms of local iron handling lung cancer can exploit to support tumorigenesis.

## Introduction

Lung cancer represents the most frequent malignant neoplasm in most countries and the leading cause of death worldwide in both sexes (1). The incidence of lung cancer is low in people aged below 40 years but it dramatically increases up to ages 60–65 years in most populations. The most common subtype of lung cancer is non-small cell lung cancer (NSCLC; 85%), the most prevalent form being lung adenocarcinoma (LUAD), followed by lung squamous cell carcinoma (LUSC) and large cell carcinoma (2). Smoking status is certainly the most important causative link in lung cancer development even though air pollution represents another paramount source of risk factor (3). Airborne Particulate matter (PM), in particular the small size components (PM_10_, PM_2.5_ and ultrafine particles-UFP), which include combustion products, soot, exhaust emission from vehicles and industrial processes, have attracted attention mainly for two reasons: firstly, due to their small size, these particles remain suspended in the air for quite a long time, thus increasing the chance of being inhaled; secondly, these particles are vehicles for chemical compounds, in particular transition metals, since iron is present in significant concentration (4). Iron is also found in cigarette smoke, the strongest causative link to pulmonary pathology (5, 6), and in asbestos fibers, which are the most frequent cause of occupational cancer (7).

Iron toxicity derives from its high redox cycling reactivity which can drive the production of free radical species (ROS) known to promote many aspects of tumor development and progression (8). The lung is extremely sensitive to metal-induced oxidative stress due to its unique role in the massive transfer of oxygen into the bloodstream (9). Therefore, as a protective strategy to prevent ROS generation, lung epithelial cells have developed a tight control on iron import, storage and export in order to keep intracellular iron concentration low, while sustaining the metabolic demand (10). Efficient iron uptake and intracellular sequestration can limit its toxicity, but if iron import exceeds the long-term storage capacity of the cell, as it occurs in iron overload conditions, the chances it may mobilize increase, resulting in oxidative stress and cell damage. Iron export mechanisms are therefore necessary to prevent excessive intracellular accumulation, as may occur when exogenous iron supplies increase as a result of airborne pollutants inhalation. The only known non-heme iron export pathway relies on the activity of the transmembrane ferrous iron transporter Ferroportin 1 (FPN1), also known as solute carrier family 40 member 1 (SLC40A1) (11), in conjunction with members of the multicopper ferroxidases family, which are required to oxidize ferrous iron to its ferric form (12). Only three multi-copper oxidases have been identified so far, namely ceruloplasmin (CP), hephaestin (HEPH) and zyklopen (ZP) (13–15). These ferroxidases promote iron transport in different tissues: HEPH is mostly expressed in the small intestine (14) but it is also present in other tissues (16); CP is mainly found as a soluble serum protein, but it is also membrane-bound *via* a glycosylphosphatidylinositol (GPI)-anchor in astrocytes and kidney (17, 18); ZP has been proposed to be involved in placental iron transport, but this has not yet been verified (19). In enterocytes FPN1, functionally associated with HEPH, allows the translocation of iron across the basolateral membrane and its release into the bloodstream (20). In the lung, instead, FPN1 is mainly expressed in the apical membrane of the airway epithelium (21) where it is believed to promote iron release into the airways or the lumen of the alveoli to meet the need for detoxification. This egress pathway has been shown to be compromised in various types of cancers (22). In particular FPN1 mRNA expression levels appeared significantly down-regulated in lung tumor, as compared to matched healthy tissue, a condition that is likely to guarantee an increase in the intracellular labile iron pool necessary for all metabolic processes involved in cell proliferation (23).

The role played by HEPH in iron metabolism in lung is still poorly characterized, and so is its possible contribution to lung carcinogenesis and growth. We recently identified a single-nucleotide polymorphism within *HEPH* gene, leading to a missense variation of this multicopper ferroxidase, which confers protection against asbestos-dependent malignant pleural mesothelioma and lung carcinoma in exposed subjects (24, 25). Moreover, in breast cancer HEPH expression has been shown to be down-regulated by the histone methyltransferase G9a, leading to changes in iron homeostasis that burst cancer growth (26).

In the current study, we examined the expression and prognostic value of HEPH expression in LUAD and LUSC patients in databases such as UALCAN, GEPIA and Kaplan–Meier plotter. Moreover, we investigated the correlation of HEPH expression with tumor-infiltrating immune and non-immune cells that characterize the tumor microenvironment, *via* Tumor Immune Estimation Resource (TIMER). Finally, we assessed the distribution of endogenous HEPH in lung cancer tissues. Taken together, these data further support the key role played by iron dysregulation in the tumor microenvironment of lung malignancies. In this context HEPH expression, if further confirmed by retrospective studies on a broader cohort of patients, could serve as a potential prognostic marker in lung cancer pathogenesis.

## Materials and Methods

### Gene Expression and Survival Analysis

Our analysis focused on the prognostic value of the *HEPH* gene in lung adenocarcinoma (LUAD) and in lung squamous cell carcinoma (LUSC). The expression level of the gene in different carcinomas was analyzed using UALCAN (http://ualcan.path.uab.edu) and GEPIA (http://gepia.cancer-pku.cn). Those tools estimate the effect of gene expression level on the patient survival, as well as being web resources for analyzing cancer transcriptome data (27, 28). We compared the differences in mRNA level between cancers and normal tissue, using genomics data from “The Cancer Genome Atlas” (TCGA lung). The prognostic significance of *HEPH* mRNA expression and survival in LUAD and LUSC were analyzed by Kaplan–Meier plotter (https://kmplot.com/analysis). The Kaplan–Meier plotter uses genomic data from the Gene Expression Omnibus and the European Genome-phenome Archive to generate survival probability plots and to perform survival analysis. The same analysis was performed for the following cell-type specific genes: *ACTA2* (α-SMA), a marker of vascular muscular cells and pericytes (29); fibroblasts activation protein (FAP), platelet-derived growth factor receptor-α/β (*PDGFRA/B*), biological markers for CAFs (30); *PECAM1* (*CD31*) and von Willebrand Factor (*vWF*) markers for endothelial cells (31, 32). The hazard ratio with 95% confidence intervals and log-rank *p*-value were also computed.

### Protein Expression Analysis

The expression of HEPH proteins between cancer and normal tissue were analyzed using UALCAN, which provides a protein expression analysis option using data from the Clinical Proteomic Tumor Analysis Consortium (CPTAC) Confirmatory/Discovery dataset (33). The *CPTAC* dataset relies on the RPPA platform, which involves micro-blots of protein lysates from multiple samples of tissues on a single array, with each sample represented by at least one spot. Each array is incubated with one specific antibody, in order to detect the relative expression of the corresponding protein across many samples simultaneously. Protein levels are quantitated by mass spectrometry-based proteomics analysis. Unfortunately, at the time of writing, the UALCAN tool only provided data for the LUAD histotype.

### TIMER Database Analysis

TIMER is a comprehensive resource for systematic analysis of immune infiltrates across diverse cancer types (www.cistrome.shinyapps.io/timer/) (34). TIMER applies a statistical method to infer the abundance of tumor-infiltrating immune cells (TIICs) from gene expression profiles using data from the TCGA dataset (35). We analyzed *HEPH* expression in lung cancers, and the correlation between its expression and the abundance of immune infiltrates, including B cells, CD4+ T cells, CD8+ T cells, neutrophils, macrophages, cancer associated fibroblasts and endothelial cells *via* gene modules. These gene markers are referenced in prior studies. Gene expression levels against tumor purity are also displayed (36, 37). Tumor purity is defined as the proportion of cancer cells present in the tumor tissue, and reflects the characteristics of tumor microenvironment. Low tumor purity is associated with a consistent recruitment of diverse kinds of tumor-infiltrating immune cells as well as stromal cells (fibroblasts, endothelial cells and pericytes). The computational algorithms of TIMER take “tumor purity” into account when analyzing a specific gene expression profile. Thus, a gene characterized by a negative association with the tumor purity parameter is expected to be expressed in cells of the microenvironment, while a gene that shows a positive correlation is expected to be mostly expressed by cancer cells. The correlation module generated the expression scatter plots between several genes and defined genes of TIICs in chosen carcinomas, together with the Spearman’s correlation and the estimated statistical significance. Several genes were used for the x-axis, and the related marker genes were represented on the y-axis as genes of TIICs. The gene expression level was displayed with log2 RSEM.

### Statistical Analysis

Survival curves were generated by the Kaplan–Meier plotter (38). All results are displayed with *p*-values from a log-rank test. *p*-values <0.05 were considered significant. In TIMER, the correlation of gene expression was evaluated by Spearman’s correlation and statistical significance, and the strength of the correlation was determined using the following guide for the absolute value: 0.00–0.19 “very weak,” 0.20–0.39 “weak,” 0.40–0.59 “moderate,” 0.60–0.79 “strong,” 0.80–1.0 “very strong”.

### Immunohistochemistry Analysis on Tumor Tissues

All lung cancer tissue samples for this study were collected according to the Helsinki Declaration and the study was approved by the University of Palermo Ethical Review Board (approval number 09/2018). A specific informed consent was not required at the time of tissue sample collection for immunohistochemical analysis of archival tissue sections, since the patients were not identified and genetic analysis was not carried out. Surgically removed malignant tissue samples, together with the adjacent non-tumor tissue, were selected for immunohistochemical analysis for HEPH expression. Invasive malignant neoplasia specimens included the two most represented histotypes including LUAD and LUSC. Tissue sections were obtained from at least ten different patients for each histotype. The study was approved by the Institutional review board of the University of Palermo (09/2018).

Immunohistochemistry was carried out on FFPE human tissue sections. Briefly, 4 micron-thick sections were cut from paraffin blocks, dried, de-waxed and rehydrated. The antigen unmasking technique was performed using Target Retrieval Solutions, pH = 6 EDTA-based buffer in thermostatic bath at 98°C for 30 min. After the sections were brought at room temperature, neutralization of endogenous peroxidase with 3% H_2_O_2_ and protein blocking by a specific protein block, were performed. For HEPH immunostaining, sections were probed with mouse monoclonal anti-human HEPH (dilution 1:100, pH 6, Clone sc-365365 Santa Cruz Biotecnology) overnight at 4°C. Antibody–Antigen recognition was detected using Novolink Polymer Detection Systems (Novocastra Leica Biosystems, Newcastle), and high sensitivity AEC (3-Amino9-Ethylcarbazole) as chromogen. Slides were counterstained with Harris Hematoxylin (Novocastra, Ltd).

For double-labeling experiments, sections were additionally probed with rabbit polyclonal anti-human CD31 (dilution 1:50, pH 9, ab28364 Abcam), rabbit monoclonal anti-human PDGFRβ (dilution 1:250, pH 6, clone Y92, ab32570 Abcam) and rabbit polyclonal anti-Ferroportin (1:1,000, pH 6, PA5-64232, Invitrogen) and anti-Myeloperoxidase antibody (1:50, pH 6, ab9535 Abcam). Staining was carried out *via* Novolink Polymer Detection Systems (Novocastra, Leica Biosystems) and DAB (3,3′ -Diaminobenzidine; Dako, Denmark) substrate-chromogen. All the sections were analyzed under Zeiss Axio Scope A1 optical microscope (Zeiss, Germany) and microphotographs were acquired using an Axiocam 503 Color digital camera with the ZEN2 imaging software (Zeiss Germany).

## Results

### The mRNA Expression Levels of HEPH in Different Types of Human Cancers

**Figure 1:**
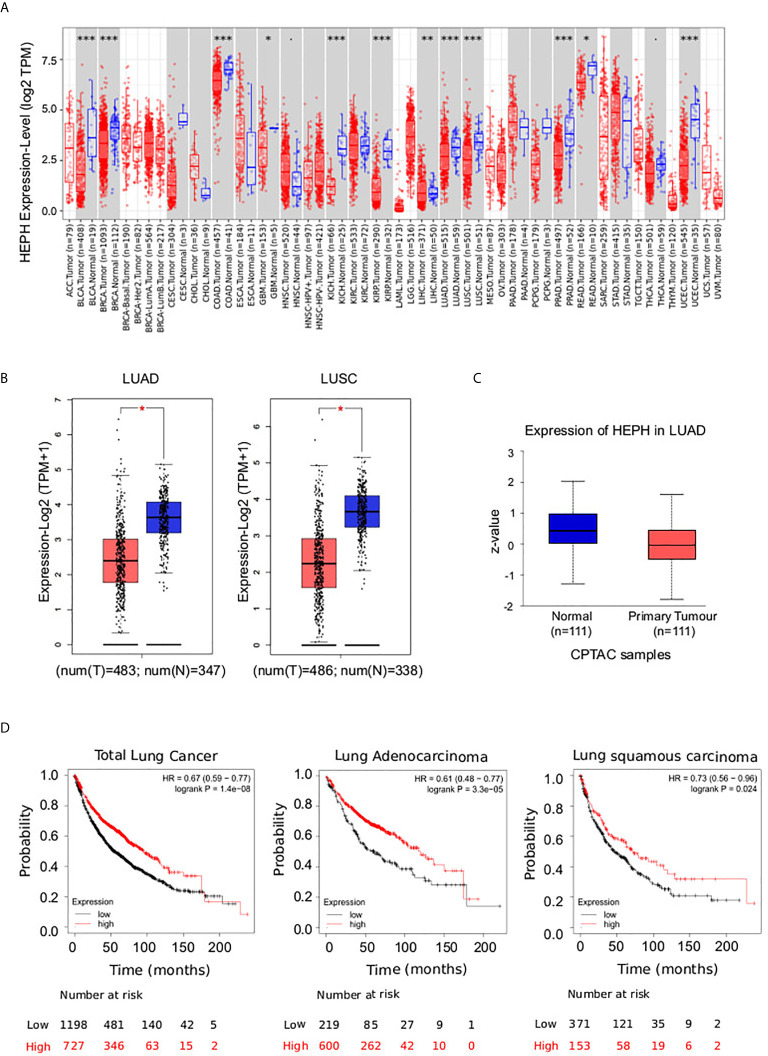
Pathological significance of HEPH expression in different types of human cancer, and in-depth evaluation in LUAD and LUSC. **(A)** Human HEPH expression levels in different tumor types from TCGA database were determined by TIMER (*P < 0.05, **P < 0.01, ***P < 0.001). **(B)** HEPH mRNA expression comparisons between normal (blue) and tumor tissues (red) obtained from the GEPIA web tool. **(C)** HEPH protein expression comparison between normal and tumor tissues obtained from the UALCAN web tool (Wilcoxon test). P-value <0.05 was used to assess differences. **(D)** Survival analyses of HEPH by Kaplan–Meier estimator with log-rank test obtained from the Kaplan–Meier plotter web tool. Survival differences are compared between patients with high (red) and low (black) HEPH expression (grouped according to Auto select best cut-off). H, high expression; L, low expression.

Ferroxidase HEPH has recently been shown to play a role in breast tumor cell growth; in particular its decreased expression has been significantly correlated with poor survival in affected patients (26). In order to expand the analysis to other cancer types, we examined HEPH expression using UALCAN to analyse TCGA RNA-sequencing and patients’ clinical data from 33 different cancer types, including several metastatic tumors (34). This analysis revealed that a significant down-regulation of HEPH mRNA expression levels is found in several other malignancies such as BLCA (bladder urothelial carcinoma), BRCA (breast invasive carcinoma), COAD (colon adenocarcinoma), KICH (kidney chromophobe), KIRP (kidney renal clear cell carcinoma), LIHC (liver hepatocellular carcinoma), LUAD (lung adenocarcinoma), LUSC (lung squamous adenocarcinoma), PRAD (prostate adenocarcinoma), READ (rectum adenocarcinoma), and UCEC (uterine corpus endometrial carcinoma) compared to the corresponding normal tissues (**Figure 1A**).

Given our interest in better understanding the role iron dysregulation may exert in lung cancer development and prognosis, we evaluated HEPH mRNA expression levels in the most prevalent histological types, LUAD and LUSC, as compared to normal tissue, utilizing the GEPIA database. Consistent with the previous analysis, a significant decrease in HEPH mRNA expression was found in LUAD and LUSC compared to healthy controls (**Figure 1B**). This reduction was confirmed at protein level only for the LUAD histotype based on the UALCAN dataset (**Figure 1C**), since correspondent proteomic data for LUSC are still not available.

To investigate the correlation between HEPH expression and patient outcome we employed the Kaplan–Meier overall survival curves to establish and compare the survival differences between patients with high and low expression of the ferroxidase (grouped according “Auto select best cutoff”) (**Figure 1D**). In both the LUAD and LUSC datasets, the high expression group had a significantly longer overall survival than the low expression group, thus indicating that higher HEPH expression correlates with better prognosis.

### HEPH Expression Is Correlated Mostly With Non-Immune Infiltration

**Figure 2:**
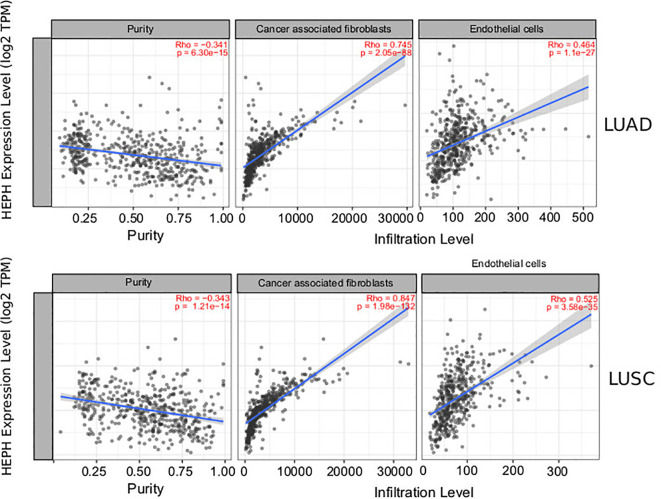
Correlation of HEPH expression with infiltration level of non-immune cells in LUAD and LUSC. HEPH expression is significantly negatively correlated to tumor purity and has significant positive correlations with the infiltrating levels of cancer-associated fibroblasts and endothelial cells.

It is well established that cancer cells are characterized by an iron-seeking phenotype, which is fundamental to support the enhanced metabolic demand characteristic of actively proliferating cells (39). The increased request in iron supply is met not only by up-regulating iron import pathways while down-regulating storage and export routes, but also by altering how other cell types of the tumor microenvironment, including immune cells, endothelial cells, pericytes and fibroblasts, metabolize iron (40, 41). We therefore investigated the correlations of HEPH expression and immune and non-immune infiltration levels, using the TIMER web resource (34). In particular, we assessed B cells, CD4+ T cells, CD8+ T cells, macrophages and dendritic cells, as immune infiltrates, while cancer associated fibroblasts and endothelial cells were analysed as infiltrating non-immune cell types. The results showed that, in both types of lung cancer, HEPH expression had a significant negative correlation with tumor purity, the parameter that identifies the proportion of cancer cells present in the tumor tissue (**Figure 2**). In addition, HEPH expression showed a very weak correlation with all infiltrating immune elements tested (**Table 1**), while a strong positive correlation was found only with cancer associated fibroblasts (CAFs) and endothelial cells (ECs) (**Figure 2**).

**Table 1:** Correlation analysis between HEPH expression and immune infiltration level of the indicated immune cells.

	LUAD		LUSC	
	*Rho*	*p*	*Rho*	*p*
Purity	−0.341	6.3e−15	−0.343	1.21e−14
CD8+ T cell	0.184	3.9e−05	0.273	1.38e−09
CD4+ T cell	0.167	2.03e−04	0.233	2.55e−07
Macrophages	0.406	5.7e−21	0.206	5.89e−06
Neutrophis	0.285	1.03e−10	0.278	6.15e−10

The “Purity Adjustment” option was applied to all analyses performed.

CAFs are the most abundant cells in solid cancer. They can be derived from several sources including activation of resident fibroblasts (42), epithelial-mesenchymal transition of epithelial cells (43), endothelial–mesenchymal transition of resident endothelial cells (44). Compared to normal fibroblasts they are characterized by enhanced proliferative and migratory features, and they are also more metabolically active. Tumor endothelial cells are the cells lining the tumor-associated blood vessels that provide nutrition and oxygen to the tumor, contributing to its growth and development. They also constitute one of the main sources of cancer-associated fibroblasts (CAFs).

**Figure 3:**
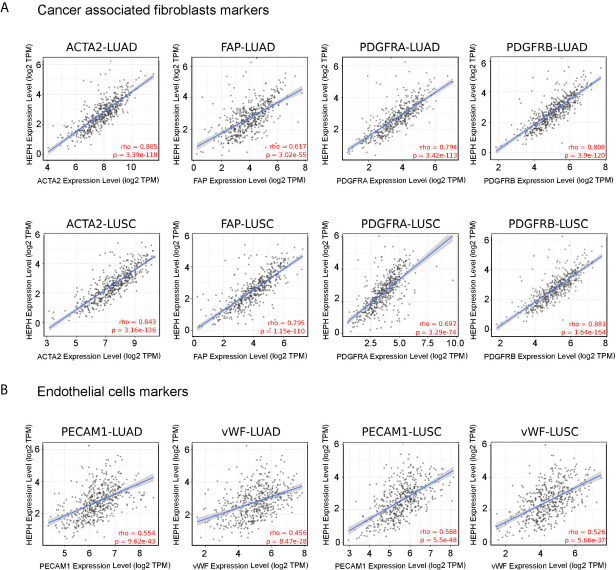
HEPH expression positively correlated with markers of cancer-associated fibroblasts **(A)** and endothelial cells **(B)** in both LUAD and LUSC. Scatterplots of correlations between HEPH and gene markers include ACTA2, FAP, PDGFRA, PDGFRB for cancer-associated fibroblasts and PECAM1 (CD31) and vWF for endothelial cells.

To further characterize the relationship between HEPH and these infiltrating cells in lung malignancies, we explore the correlation between HEPH and a list of marker sets known to be widely used to identified CAFs and ECs, using the TIMER Gene Correlation module. In particular, we used α-SMA (ACTA2, also marker for vascular muscular cells and pericytes), fibroblasts activation protein (FAP, also expressed in a subset of CD45+ immune cells), and platelet-derived growth factor receptor-α/β (PDGFRA/B) as biological markers for CAFs (**Figure 3A**); PECAM1 (CD31) and von Willebrand Factor (vWF) as markers for endothelial cells (**Figure 3B**). After adjusting the correlation by tumor purity, HEPH expression level was significantly correlated with all tested marker sets (**Figure 3**).

**Figure 4:**
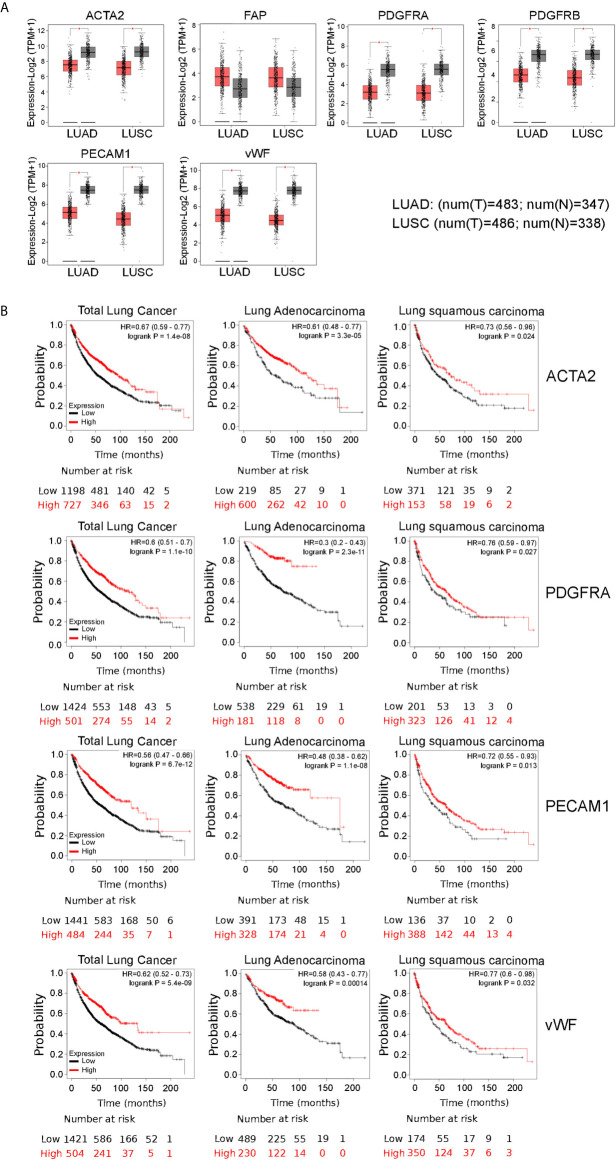
Comparisons of the mRNA expression levels of cancer-associated fibfroblasts and endothelial cells markers between normal (grey) and tumor tissues (red) **(A)**. Overall survival curve of each cancer-associated fibroblasts and endothelial marker shown to correlate with HEPH expression and produced by Kaplan–Meier website resource **(B)**. OS differences are compared between patients with high and low HEPH expression (grouped according to Auto select best cut-off). H, high expression; L, low expression.

Interestingly, we also found that the mRNA expression level of all these marker genes, with the only exception of FAP, were significantly down-regulated in both lung malignancies, as compared to paired normal tissues, based on GEPIA datasets (**Figure 4A**). Moreover, Kaplan–Meier analysis indicated that high expression of ACTA2 and PDGFRA, as well as PECAM1 and vWF, was associated with better overall survival, as it is for HEPH expression (**Figure 4B**).

### Distribution of HEPH in Clinical LUAD and LUSC Specimens

**Figure 5:**
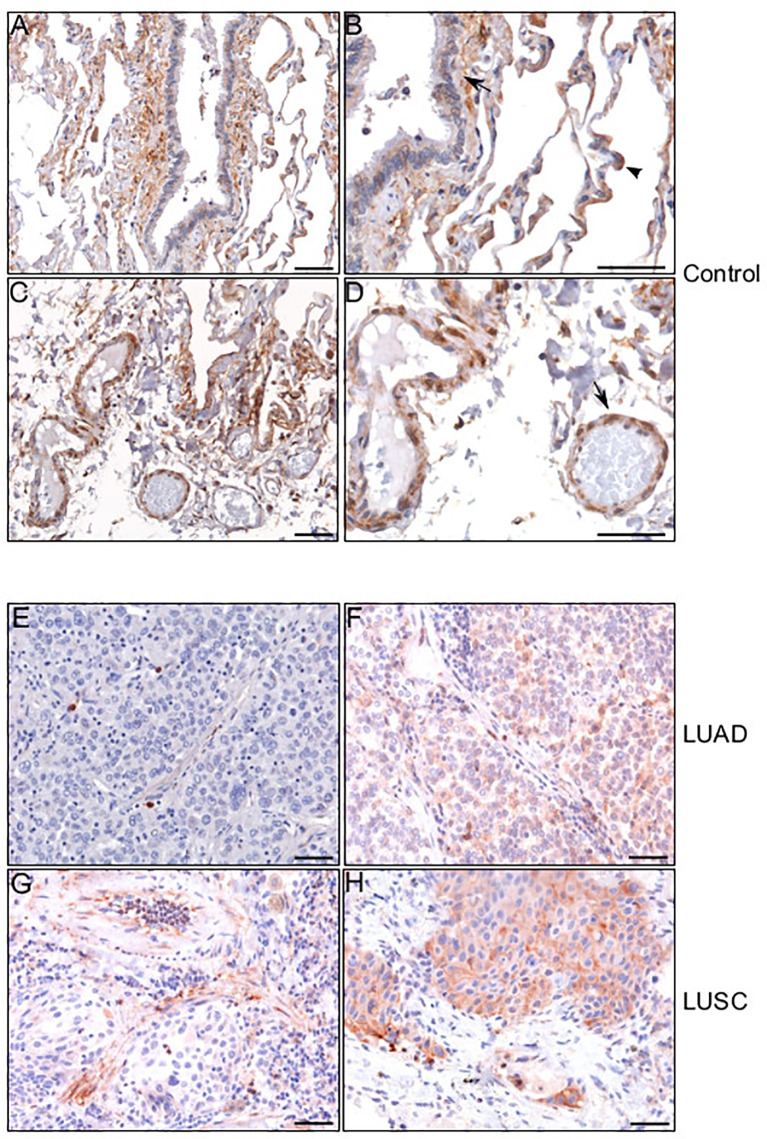
HEPH distribution in control non-tumor lung and in LUAD and LUSC specimens. Representative microphotographs relative to HEPH distribution in non-tumor lung **(A–D)**. In panel **(B)** an HEPH expressing type II pneumocyte is indicated by arrow-head while a black arrow points to smooth muscle fibers and bronchiolar epithelium. In panel **(D)** endothelial cells are indicated by an arrow. HEPH distribution by cancer cells in the context of the two histotypes. Panels **(E)** (LUAD) and **(G)** (LUSC) show the tumoral areas in which HEPH is poorly or not expressed. Panels **(F)** (LUAD) and **(H)** (LUSC) correspond to cancer nests expressing HEPH. Polymer detection system with AEC (red) chromogen; scale bars, 50 µm.

Based on the results obtained from the TIMER database analysis, we set out to better understand the distribution of HEPH in a series of specimens of LUAD and LUSC upon ferroxidase immunohistochemical labeling. Immunolocalization on normal lung specimes showed that HEPH was expressed by several cell types (**Figures 5A, C**): the epithelial cells of the alveoli, mainly type II pneumocytes, identified based on their round-shape morphology (**Figure 5B**, arrow-head); the epithelial cells of the bronchiole together with the smooth muscle fibers surrounding the bronchiolar epithelium (**Figure 5B**, black arrow); the endothelial cells of the micro vessels (**Figures 5C, D**). HEPH was mainly observed in the cytoplasm.

**Figure 6:**
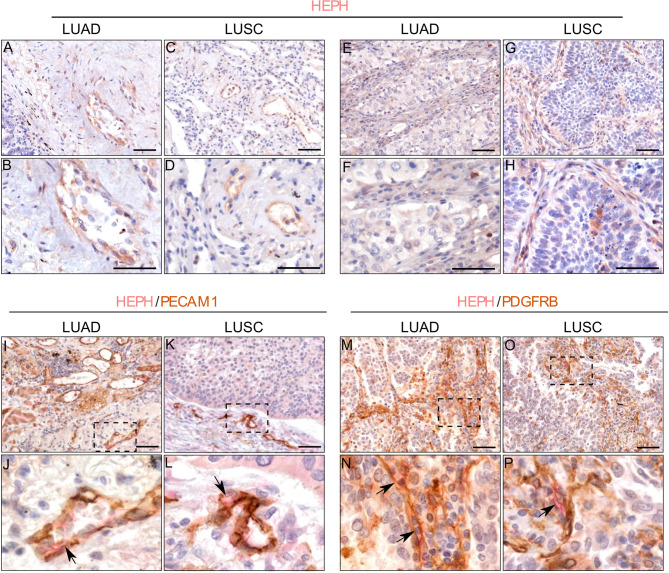
HEPH expression by endothelial and stromal cells in LUAD and LUSC specimens. Endothelial cells identified morphologically in single HEPH immunostaining (panels **(A, B)** for LUAD and **(C, D)** for LUSC), and by way of the strong expression of PECAM1 (CD31) upon double-labeling (panels **(I, J)** for LUAD and **(L, M)** for LUSC). Black arrows indicate HEPH/PECAM1 colocalization. Fibroblasts identified based on their spindle-shaped morphology in single HEPH immunostaining [panels **(E, F)** for LUAD and **(G, H)** for LUSC], and by the expression of PDGFR-β upon double-labeling. Back arrows indicate HEPH/PDGFR-β colocalization [panels **(M, N)** for LUAD and **(O, P)** for LUSC]. Panels **(J, M, N, P)** represent higher magnifications of the corresponding dashed area indicated on the corresponding upper panel. Polymer detection system with AEC (red) chromogen for HEPH and DAB (3,3′-Diaminobenzidine) chromogen for PECAM1; scale bars, 50 µm.

HEPH distribution in cancer tissues, appeared to be quite different in the two malignancies. Cancer cells in most of the analyzed LUAD specimens were totally lacking HEPH (**Figure 5E**), even though a few clumps of neoplastic cells surrounded by stroma, the so-called tumor nests (**Figure 5F**), positive to HEPH labeling, could be observed. In LUSC, instead, approximately 30% of cancer cells, identifiable by their characteristic large polygonal shape, expressed the ferroxidase to variable extent (**Figures 5G, H**). Cancer cells HEPH staining was mainly cytosolic but, in some cases, also it was also clearly detected at the cell membrane (**Supplemental Figure 1A**). In both malignancies, HEPH expression was quite intense on the vascular endothelium in the peri-tumoral tissues (**Figures 6A, B**, LUAD and LUSC panels **C**, **D**), as identified by PECAM1 (also known as CD-31)/HEPH double-labeling immunoreactivities (**Figures 6I, J**, LUAD panels and LUSC panels **K, L**).

Moreover, the tumor masses in LUSC and LUAD were also characterized by the presence of HEPH positive mesenchymal cells exhibiting spindle-shaped morphology, reminiscent of fibroblastic stromal component (**Figures 6E, F**, LUAD and LUSC panels **G, H**). In double-labeling experiments only few of these stromal cellular elements co-labeled with PDGFRβ, a recognized marker for CAFs (**Figures 6M, N**, LUAD and LUSC panels **O, P**).

**Figure 7:**
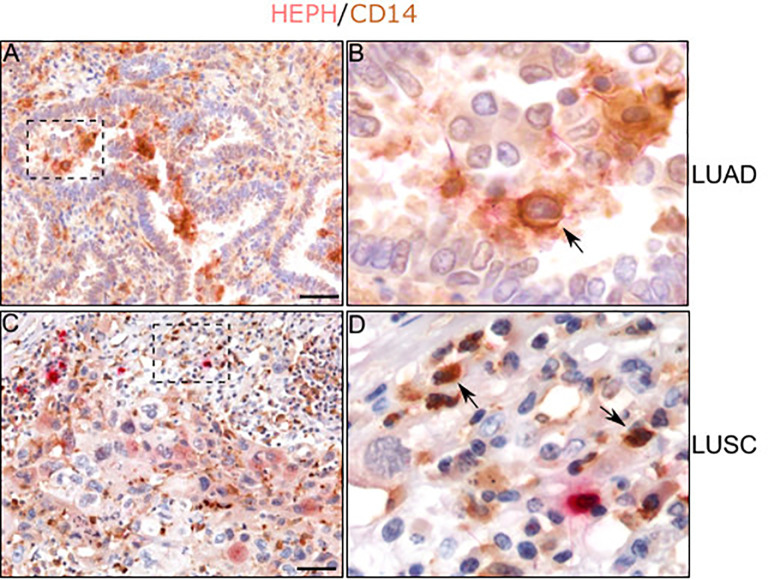
HEPH distribution in tumor-associated macrophages. Representative microphotographs relative to HEPH expression in monocytes/macrophages recognized by CD14 immunoreactivity in LUAD **(A, B)** and LUSC **(C, D)**. Black arrows indicate HEPH/CD14 colocalization. Polymer detection system with AEC (red) chromogen for HEPH and DAB (3,3′ -Diaminobenzidine) chromogen for CD14; scale bars, 50 µm.

Regarding immune infiltrates, in both histotypes we observed the presence of some HEPH expressing monocyte/macrophages, identified by their spherical appearance and their positivity for the marker CD14, a glycolipid-anchored membrane glycoprotein expressed on cells of the myelomonocyte lineage (**Figure 7**, see arrows). On the contrary, neutrophils, identified by their small round shape, the presence of a clearly identifiable multi-lobed nucleus and by their positivity for the marker MPO, were only occasionally found positive for HEPH staining, in both LUAD and LUSC specimens (**Supplemental Figure 1B**).

Overall, the immune-labeling experiments, in concordance with the bioinformatics analysis, confirm the hypothesis that HEPH is expressed mostly by endothelial cells and stromal elements infiltrating the tumor microenvironment.

## Discussion

Lung cancer still represents the leading cause of cancer-related deaths both in men and in women, especially in developed countries (45, 46). Lung adenocarcinoma is the most common histologic subtype, its incidence having risen dramatically, surpassing in fact that of squamous cell carcinoma, due to the increased incidence of lung cancer in women (47). Despite advances in diagnosis and treatments, the overall 5-year survival rate remains dismal, especially when lung cancer is diagnosed at advanced stages (48). Therefore, a better understanding of the molecular mechanisms underlying lung carcinogenesis could contribute to the development of novel strategies for prevention and therapy. Cigarette smoking represents the main risk factor for lung cancer, however, inhalation of iron-rich air pollution particles (49) as well as asbestos fibers, even if with a lower risk factor (50), are also accountable for the increased incidence of this type of malignancies. Air pollution and tobacco smoking have been shown to impact on lung iron metabolism, increasing iron supply in a tissue that is physiologically exposed to oxidative stress. In the present study, supported by bioinformatic evidence, we identified HEPH, a protein involved in exporting iron out of the cell, as a promising predictor of clinical prognosis in lung cancer.

HEPH is a multi-copper oxidase whose function has been better characterized for the small intestine, where it is required for iron egress from the enterocyte into the circulatory system (14, 51). HEPH has been shown to act in concert with Ferroportin (FPN1) (52), the only known mammalian iron exporter for non-heme iron, the mRNA down-regulation of which has been detected in several cancers, usually correlated to poor prognosis (23). In the healthy lung, FPN1 is facing the lumen of the alveoli and this localization has been associated to a role in iron detoxification (21). Indeed, environmental iron reaching the lung epithelium can initially be buffered by the activity of antioxidant molecules such as ascorbic acid, reduced glutathione, and mucin. Once loaded on the transferrin and lactoferrin herein present, it can undergo transferrin receptor 1 (TfR1) and lactoferrin receptor (LfR) internalization by epithelial alveolar cells (53) and alveolar macrophages, and be stored safely, bound to ferritin (54). Under conditions of iron overload, excess pulmonary iron can be released into the lumen of the alveoli *via* FPN1 permease, and possibly oxidized by GPI-anchored or soluble ceruloplasmin, a ferroxidase homologous to HEPH (55).

In the context of cancer, the observed reduction in FPN1 is expected to increase the concentration of the intracellular iron pool, a condition required to sustain the high metabolic demand of actively proliferating cells. Based on bioinformatic evidence, also HEPH mRNA expression levels are downregulated in several malignancies, including lung cancer and, similarly to FPN1, such down-regulation correlates with poor prognosis. Interestingly, HEPH/FPN1 double-labeling experiments showed that both ferroxidase and its functionally-coupled iron permease were both poorly expressed in most of the cancer cells of the analysed LUAD and LUSC specimens, while their expression was still maintained in nesting arrangement of cancer cells having a characteristic epithelial differentiation (**Supplemental Figure 2**). Tumor cell differentiation status is a very important aspect; it is scored and evaluated for clinical diagnosis since it correlates with tumor aggressiveness and worse prognosis (56). The fact that a higher expression of HEPH/FPN1 partners is detected in still well-differentiated cancer cell nests may prove their ability to correctly handle iron. By conferring a better prognosis, this feature could make HEPH expression a relevant prognostic marker to predict a patient’s clinical course

Our study has also shown that HEPH is expressed by the endothelial cells of the lung vasculature in the peri-tumoral tissue of both histotypes. To our knowledge, this peculiar HEPH distribution is only seen in the capillaries of the central nervous system, where brain microvascular endothelial cells, in association with astrocytes and pericytes, exert a tight control on iron entry into the brain (57). In this context, the ferroxidase has been shown to localize on the endothelium abluminal side, where it is presumed to convert ferrous iron, released in the extracellular space by endothelial FPN1, into ferric iron, thus limiting the oxidative damage. HEPH/FPN1 double labeling experiments demonstrated a partial co-localization of the two markers on endothelial cells belonging to the blood vessels situated close to the cancerous mass (**Supplemental Figures 3A**, panels **A, B**). Taken together, these data would support the notion that iron flux in the lung could operate similarly to what has been described for the brain, with endothelial-localized HEPH assisting FPN1 in shipping nutritional ferrous iron into the interstitial space, making it available for resident cell uptake.

TIMER bioinformatics identified a strong positive correlation between HEPH expression and cancer associated fibroblasts (CAFs). These cells are the most dominant cellular component in the tumor stroma,. They not only provide physical support to tumor cells but also play key role in promoting or hampering tumorigenesis in a context-dependent manner. CAFs are tremendously heterogeneous in phenotype, function and prognostic significance (58, 59) and can originate from resident fibroblasts, bone marrow-derived progenitor cells or epithelial/endothelial cells that have undergone epithelial to mesenchymal transition (60). Through HEPH immune-labeling of LUAD and LUSC specimens, we clearly identified HEPH-expressing cells, characterized by the typical elongated spindle-shaped morphology of fibroblasts, enveloping some tumor nests in both LUAD and LUSC cancer histotypes. A subpopulation of these cellular elements also co-labeled with PDGFRβ, a key regulator of mesenchymal cell activity in the tumor microenvironment (61), while most of them were negative for α-smooth muscle actin (α-SMA) expression (data not shown). Heterogeneity was further underlined by the observed variable degree on Ferroportin/HEPH co-labeling (**Supplemental Figures 3A**, panels **C, D**), thus increasing the complexity of the scenario. It is interesting to note that a recent study identified at least seven diverse subpopulations of fibroblasts in lung cancer, varying in abundance between cancer subtypes, and shown to accumulate in spatially distinct niches, possibly associated to achieve functional synergy (62). Our results introduce an additional layer of complexity by highlighting the multifaced and interconnected ways in which each cell type tailors iron handling to fulfil its own needs. An aspect, this, that requires further investigations.

Finally, our study underscored the presence of CD14 positive monocyte/macrophages, expressing HEPH to different extents, in all LUAD and LUSC specimens analyzed. Tumor associated macrophages (TAMs) are found in most malignancies, where they facilitate angiogenesis, remodelling of the extracellular matrix, tumor cell invasion and migration while suppressing immune-response (63). TAMs are characterized by an iron-release phenotype achieved by lowering the expression of the iron storage protein ferritin, while increasing the expression of the only iron exporter FPN1 (64). Based on double labeling experiments, we observed that HEPH expressing TAMs were mostly colocalizing with FPN1 immuno-reactivity (**Supplemental Figure 3B**), thus supporting their possible role as iron suppliers for tumor cells.

In conclusion, our results further underline the complex, and still poorly understood, association that exists between iron metabolism and the cancerogenic mechanisms operating in different organ landscapes. Bioinformatic analysis based on mRNA expression dataset, indicates HEPH as a potential novel prognostic biomarker for lung cancer pathologies. Up-regulation of HEPH in LUAD and LUSC correlates with a better outcome since, in association with ferroportin activity, it’s expected to avoid the increase of the intracellular concentration of free iron, known to promote cell proliferation. The novelty of our study lays in having shown that HEPH, together with FPN1, resides mainly on stromal cellular elements, in particular endothelial cells and fibroblasts, key players in the tumorigenic process. Despite the limitations of our immunohistochemical characterization of HEPH distribution in LUAD and LUSC histotypes, which requires further validation on a broader cohort of patients, the current findings illustrate how complex, multifaced and still poorly understood, is the contribution of iron handling in the pathogenesis of lung cancer. In fact, only upon gaining a full understanding of the functional cross-talk that occurs between the different cell types, HEPH-expressing cells and cancer cells, will it be possible to envisage a clinical use for HEPH as a prognostic marker, exploiting it as new therapeutic target to fight these devastating diseases.

## Data Availability Statement

The raw data supporting the conclusions of this article will be made available by the authors, without undue reservation.

## Ethics Statement

The studies involving human participants were reviewed and approved by University of Palermo Ethical Review Board (approval number 09/2018). Written informed consent for participation was not required for this study in accordance with the national legislation and the institutional requirements.

## Author Contributions

Conception and design: PZ, AM, and VB. Development of methodology: PZ and AlM. Acquisition of data: BB, LS, GM, and AnM. Analysis and interpretation of data (e.g., statistical analysis, biostatistics, and computational analysis): AlM, PZ, and VB. Writing, review, and/or revision of the manuscript: PZ, AlM, and VB. Study supervision: VB. All authors contributed to the article and approved the submitted version.

## Funding

This work was supported by grants from the Italian League for the Fight Against Cancer (LILT), Gorizia section (Bando di Ricerca sanitaria 2017-programma 5 per mille anno 2015).

## Conflict of Interest

The authors declare that the research was conducted in the absence of any commercial or financial relationships that could be construed as a potential conflict of interest.

## Supplementary Material

Supplementary Figure 1
**(A)** HEPH is clearly detected at the cell membrane is some neoplastic nests **(**panel **A)**. A higher magnification of the dashed area is reported on panel B. **(B)** HEPH is not expressed by neutrophils. Representative microphotographs relative to lack of HEPH expression in neutrophils recognized by MPO immunoreactivity (indicated by arrows) in LUAD **(A, B)** and LUSC **(C, D)**. Polymer detection system with AEC (red) chromogen for HEPH an d DAB (3,3′ -Diaminobenzidine) chromogen for CD14; scale bars, 50µm.

Supplementary Figure 2 HEPH co-expresses with FPN1 in some cancer nests. Representative microphotographs relative to HEPH/FPN1 co-expression in two cases of LUAD **(A, B)** and LUSC **(C, D)**. Polymer detection system with AEC (red) chromogen for HEPH an d DAB (3,3′ -Diaminobenzidine) chromogen for CD14; scale bars, 50µm.

Supplementary Figure 3 HEPH/FPN1 are partially co-expressed on endothelial cells and stromal fibbroblasts, as well as on macrophages. **(A)** Representative microphotographs relative to HEPH/FPN1 co-expression on endothelial cells **(A, B)** and fibroblasts **(C, D)** (see black arrows). **(B)** Representative microphotographs relative to HEPH/FPN1 co-expression on macrophages in LUAD **(A, B)** and LUSC **(C, D)** (see black arrows). Polymer detection system with AEC (red) chromogen for HEPH an d DAB (3,3′ -Diaminobenzidine) chromogen for CD14; scale bars, 50µm.

## References

[1] Barta JA Powell CA Wisnivesky JP. Global Epidemiology of Lung Cancer Classifications. Ann Glob Health (2019) 85:1–16. 10.5334/aogh.2419 30741509 PMC6724220

[2] Inamura K. Lung Cancer: Understanding its Molecular Pathology and the 2015 WHO Classification. Front Oncol (2017) 7:193. 10.3389/fonc.2017.00193 28894699 PMC5581350

[3] Zhou G. Tobacco, Air Pollution, Environmental Carcinogenesis, and Thoughts on Conquering Strategies of Lung Cancer. Cancer Biol Med (2019) 16(4):700–13. 10.20892/j.issn.2095-3941.2019.0180 PMC6936241 31908889

[4] Lorelei de Jesus A Rahman M Mazaheri M Thompson H Knibbs LD Jeong C. Ultrafine Particles and PM2.5 in the Air of Cities Around the World: Are They Representative of Each Other? Environ Int (2019) 129:118–35. 10.1016/j.envint.2019.05.021 31125731

[5] Mussala-Rauhammae H Salmela SS Lepannen A Pyysalo H. Cigarettes as a Source of Some Trace and Heavy Metals and Pesticides in Man. Arch Environ Health (1986) 41:49–55. 10.1080/00039896.1986.9935765 3963887

[6] Thompson AB Bohling T Heires A Lindner J Rennard SI. Lower Respiratory Tract Iron Burden Is Increased in Association With Cigarette Smoking. J Lab Clin Med (1991) 117:493–9. 2045717

[7] Toyokuni S. Iron Overload as a Major Targetable Pathogenesis of Asbestos-Induced Mesothelial Carcinogenesis. Redox Rep (2014) 19(1):1–7. 10.1179/1351000213Y.0000000075 24257681 PMC6837658

[8] Chen Y Fan Z Yang Y Gu C. Iron Metabolism and its Contribution to Cancer. Int J Oncol (2019) 54:1143–54. 10.3892/ijo.2019.4720 30968149

[9] Cloonan SM Mumby S Adcock IM Choi AMK Chung KF Quimlan GJ. The “Iron”-Y of Iron Overload and Iron Deficiency in Chronic Obstructive Pulmonary Disease. Am J Respir Crit Care Med (2017) 196:1103–12. 10.1164/rccm.201702-0311PP PMC5694836 28410559

[10] Ghio AJ. Disruption of Iron Homeostasis and Lung Disease. Biochim Biophys Acta (2009) 1790:731–9. 10.1016/j.bbagen.2008.11.004 19100311

[11] Ward D Kaplan J. Ferroportin-Mediated Iron Transport: Expression and Regulation. Biochim Biophys Acta (2012) 1823(9):1426–33. 10.1016/j.bbamcr.2012.03.004 PMC3718258 22440327

[12] Vashchenko G MacGillivray RTA. Multi-Copper Oxidases and Human Iron Metabolism. Nutrients (2013) 5(7):2289–313. 10.3390/nu5072289 PMC3738974 23807651

[13] Healy J Tipton K. Ceruloplasmin and What it Might do. J Neural Transm (2007) 114(6):777–81. 10.1007/s00702-007-0687-7 17406962

[14] Vulpe CD Kuo YM Murphy TL Cowley L Askwith C Libina N. Hephaestin, a Ceruloplasmin Homologue Implicated in Intestinal Iron Transport, Is Defective in the Sla Mouse. Nat Genet (1999) 21:195–9. 10.1038/5979 9988272

[15] Chen H Attieh ZK Syed BA Kuo YM Stevens V Fuqua BK. Identification of Zyklopen, a New Member of the Vertebrate Multicopper Ferroxidase Family, and Characterization in Rodents and Human Cells. J Nutr (2010) 140:1728–35. 10.3945/jn.109.117531 PMC2937573 20685892

[16] Hudson D Curtis SB Smith VC Griffiths TA Wong AY Scudamore CH. Human Hephaestin Expression is Not Limited to Enterocytes of the Gastrointestinal Tract But Is Also Found in the Antrum, the Enteric Nervous System, and Pancreatic {Beta}-Cells. Am J Physiol Gastrointest liver Physiol (2010) 298:G425–32. 10.1152/ajpgi.00453.2009 20019163

[17] Patel BN David S. A Novel Glycosylphosphatidylinositol-Anchored Form of Ceruloplasmin Is Expressed by Mammalian Astrocytes. J Biol Chem (1997) 272(32):20185–90. 10.1074/jbc.272.32.20185 9242695

[18] Fortna RR Watson HA Nyquist SE. Glycosyl Phosphatidylinositol-Anchored Ceruloplasmin Is Expressed by Rat Sertoli Cells and Is Concentrated in Detergent-Insoluble Membrane Fractions. Biol Reprod (1999) 61(4):1042–9. 10.1095/biolreprod61.4.1042 10491642

[19] Danzeisen R Fosset C Chariana Z Page K David S McArdle HJ. Placental Ceruloplasmin Homolog Is Regulated by Iron and Copper and Is Implicated in Iron Metabolism. Am J Physiol Cell Physiol (2002) 282(3):C472–8. 10.1152/ajpcell.00019.2001 11832331

[20] McKie AT Marciani P Rolfs A Brennan K Wehr K Barrow D. A Novel Duodenal Iron-Regulated Transporter, IREG1, Implicated in the Basolateral Transfer of Iron to the Circulation. Mol Cell (2000) 5:299–309. 10.1016/s1097-2765(00)80425-6 10882071

[21] Yang F Haile DJ Wang X Dailey LA Stonehuerner JG Ghio AJ. Apical Location of Ferroportin 1 in Airway Epithelia and Its Role in Iron Detoxification in the Lung. Am J Physiol Lung Cell Mol Physiol (2005) 289:L14–23. 10.1152/ajplung.00456.2004 15749737

[22] Drakesmith H Nemeth E Ganz T. Ironing Out Ferroportin. Cell Metab (2015) 22:P777–787. 10.1016/j.cmet.2015.09.006 PMC4635047 26437604

[23] Pinnix ZK Miller LD Wang W D’Agostino R Kute T Willingham MC. Ferroportin and Iron Regulation in Breast Cancer Progression and Prognosis. Sci Transl Med (2010) 2:43ra56. 10.1126/scisignal.3001127 PMC3734848 20686179

[24] Crovella S Bianco AM Vuch J Zupin L Moura RR Trevisan E. Iron Signature in Asbestos-Induced Malignant Pleural Mesothelioma: A Population-Based Autopsy Study. J Toxicol Environ Health A (2016) 79:129–41. 10.1080/15287394.2015.1123452 26818092

[25] Celsi F Crovella S Moura RR Schneider M Vita F Finotto L. Pleural Mesothelioma and Lung Cancer: The Role of Asbestos Exposure and Genetic Variants in Selected Iron Metabolism and Inflammation Genes. J Toxicol Environ Health A (2019) 82(20):1–15. 10.1080/15287394.2019.1694612 31755376

[26] Wang Y Zhang J Su Y Shen Y Jiang D Hou Y. G9a Regulates Breast Cancer Growth by Modulating Iron Homeostasis Through the Repression of Ferroxidase Hephaestin. Nat Commun (2017) 8(1):274. 10.1038/s41467-017-00350-9 28819251 PMC5561105

[27] Chandrashekar DS Bashel B Balasubramanya SAH Creighton CJ Ponce-Rodriguez I Chakravarthi BVSK. Ualcan: A Portal for Facilitating Tumor Subgroup Gene Expression and Survival Analyses. Neoplasia (2017) 19:649–58. 10.1016/j.neo.2017.05.002 PMC5516091 28732212

[28] Tang Z Li C Kang B Gao G Li C Zhang Z. GEPIA: A Web Server for Cancer and Normal Gene Expression Profiling and Interactive Analyses. Nucleic Acids Res (2017) 45:W98–02. 10.1093/nar/gkx247 28407145 PMC5570223

[29] Verbeek MM Otte-Höller I Wesseling P Ruiter DJ de Waal RM. Induction of Alpha-Smooth Muscle Actin Expression in Cultured Human Brain Pericytes by Transforming Growth Factor-Beta 1. Am J Pathol (1994) 144(2):372–82. PMC1887139 8311120

[30] Nurmik M Ullmann P Rodriguez F Haan S Letellier E. In Search of Definitions: Cancer-Associated Fibroblasts and Their Markers. Int J Cancer (2020) 146(4):895–905. 10.1002/ijc.32193 30734283 PMC6972582

[31] Lertkiatmongkol P Liao D Mei,H Hu Y Newmana PJ. Endothelial Functions of PECAM-1 (Cd31). Curr Opin Hematol (2016) 23(3):253–9. 10.1097/MOH.0000000000000239 PMC4986701 27055047

[32] Zanetta L Marcus SG Vasile J Dobryansky M Cohen H Shamamian KEP. Expression of Von Willebrand Factor, an Endothelial Cell Marker, Is Up-Regulated by Angiogenesis Factors: A Potential Method for Objective Assessment of Tumor Angiogenesis. Int J Cancer (2000) 85(2):281–8. 10.1002/(sici)1097-0215(20000115)85:2<281::aid-ijc21>3.0.co;2-3 10629090

[33] Edwards NJ Oberti M Thangudu RR Cai S McGarvey PB Jacob S. The CPTAC Data Portal: A Resource for Cancer Proteomics Research. J Proteome Res (2015) 6:2707–13. 10.1021/pr501254j 25873244

[34] Li T Fan J Wang B Traugh N Chen Q Liu JS. TIMER2.0 for Analysis of Tumor-Infiltrating Immune Cells. Nucleic Acids Res (2020) 48:W509–14. 10.1093/nar/gkaa407 PMC7319575 32442275

[35] Weinstein JN Collisson EA Mills GB Shaw KM Ozenberger BA Shmulevich KEI. And Cancer Genome Atlas Research Network. The Cancer Genome Atlas Pan-Cancer Analysis Project. Nat Genet (2013) 45:1113–20. 10.1038/ng.2764 PMC3919969 24071849

[36] An Y Liu F Chen Y Yang Q. Crosstalk Between Cancer-Associated Fibroblasts and Immune Cells in Cancer. J Cell Mol Med (2020) 24:13–24. 10.1111/jcmm.14745 31642585 PMC6933413

[37] Bussard KM Mutkus L Stumpf K Gomez-Manzano C Marini FC. Tumor-Associated Stromal Cells as Key Contributors to the Tumor Microenvironment. Breast Cancer Res (2016) 18:84. 10.1186/s13058-016-0740-2 27515302 PMC4982339

[38] Nagy A Lánczky A Menyhárt O Győrffy B. Validation of miRNA Prognostic Power in Hepatocellular Carcinoma Using Expression Data of Independent Datasets. Sci Rep (2018) 8:9227. 10.1038/s41598-018-29514-3 29907753 PMC6003936

[39] Torti SV Manz DH Paul BT Blanchette-Farra N Torti FM. Iron and Cancer. Annu Rev Nutr (2018) 38:97. 10.1146/annurev-nutr-082117-051732 30130469 PMC8118195

[40] Pfeifhofer-Obermair C Tymoszuk P Petzer Weiss G Nairz M. Iron in the Tumor Microenvironment—Connecting the Dots. Front Oncol (2018) 8:549. 10.3389/fonc.2018.00549 30534534 PMC6275298

[41] Vela D. Iron in the Tumor Microenvironment. Adv Exp Med Biol (2020) 1259:39–51. 10.1007/978-3-030-43093-1_3 32578170

[42] Fukino K Shen L Matsumoto S Morrison CD Mutter GL Eng C. Combined Total Genome Loss of Heterozygosity Scan of Breast Cancer Stroma and Epithelium Reveals Multiplicity of Stromal Targets. Cancer Res (2004) 64:7231–6. 10.1158/0008-5472.CAN-04-2866 15492239

[43] Petersen OW Nielsen HL Gudjonsson T Villadsen R Rank F Niebuhr E. Epithelial to Mesenchymal Transition in Human Breast Cancer can Provide a Nonmalignant Stroma. Am J Pathol (2003) 162:391–402. 10.1016/S0002-9440(10)63834-5 12547698 PMC1851146

[44] Piera-Velazquez S Jimenez SA. Endothelial to Mesenchymal Transition: Role in Physiology and in the Pathogenesis of Human Diseases. Physiol Rev (2019) 99(2):1281–324. 10.1152/physrev.00021.2018 PMC6734087 30864875

[45] Zappa C Mousa SA. Non-Small Cell Lung Cancer: Current Treatment and Future Advances. Transl Lung Cancer Res (2016) 5:288–300. 10.21037/tlcr.2016.06.07 27413711 PMC4931124

[46] Bray F Ferlay J Soerjomataram I Siegel RL Torre LA Jemal A. Global Cancer Statistics 2018: GLOBOCAN Estimates of Incidence and Mortality Worldwide for 36 Cancers in 185 Countries. CA A Cancer J Clin (2018) 68:394–424. 10.3322/caac.21492 30207593

[47] Lortet-Tieulent J Soerjomataram I Ferlay J Rutherford M Weiderpass E Bray F. Nternational Trends in Lung Cancer Incidence by Histological Subtype: Adenocarcinoma Stabilizing in Men But Still Increasing in Women. Lung Cancer (2014) 84(1):13–22. 10.1016/j.lungcan.2014.01.009 24524818

[48] Saab S Zalzale H Rahal Z Khalifeh Y Sinjab A Kadara H. Insights Into Lung Cancer Immune-Based Biology, Prevention, and Treatment. Front Immunol (2020) 11:159. 10.3389/fimmu.2020.00159 32117295 PMC7026250

[49] Neves J Haider T Gassmann M Muckenthaler MU. Iron Homeostasis in the Lungs-A Balance Between Health and Disease. Pharmaceuticals (2019) 12(1):5. 10.3390/ph12010005 PMC6469191 30609678

[50] Kamp DW. Asbestos-Induced Lung Diseases: An Update. Transl Res (2009) 153(4):143–52. 10.1016/j.trsl.2009.01.004 PMC3544481 19304273

[51] Fuqua BK Lu Y Frazer DM Darshan D Wilkins SJ Dunn L. Severe Iron Metabolism Defects in Mice With Double Knockout of the Multicopper Ferroxidases Hephaestin and Ceruloplasmin. Cell Mol Gastroenterol Hepatol (2018) 6:405–27. 10.1016/j.jcmgh.2018.06.006 PMC6120670 30182051

[52] Yeh KY Yeh M Glass J. Interactions Between Ferroportin and Hephaestin in Rat Enterocytes are Reduced After Iron Ingestion. Gastroenterology (2011) 141(1):292–9, 299.e1. 10.1053/j.gastro.2011.03.059 21473866

[53] Ghio AJ Carter JD Dailey LA Devlin RB Samet JM. Respiratory Epithelial Cells Demonstrate Lactoferrin Receptors That Increase After Metal Exposure. Am J Physiol (1999) 276:L933–40. 10.1152/ajplung.1999.276.6.L933 10362717

[54] Zhang V Nemeth E Kim A. Iron in Lung Pathology. Pharmaceuticals (2019) 12:30. 10.3390/ph12010030 PMC6469192 30781366

[55] Harris ZL Durley AP Tk M Gitlin JD. Targeted Gene Disruption Reveals an Essential Role for Ceruloplasmin in Cellular Iron Efflux. Proc Natl Acad Sci USA (1999) 96:10812–7. 10.1073/pnas.96.19.10812 PMC17965 10485908

[56] Jögi A Vaapil M Johansson M Påhlman S. Cancer Cell Differentiation Heterogeneity and Aggressive Behavior in Solid Tumors. Ups J Med Sci (2012) 117(2):217–24. 10.3109/03009734.2012.659294 PMC3339553 22376239

[57] Burkhart A Skjorringe T Johnsen KB Siupka P Thomsen LB Nielsen MS. Expression of Iron-Related Proteins At the Neurovascular Unit Supports Reduction and Reoxidation of Iron From Transport Through the Blood-Brain Barrier. Mol Neurobiol (2016) 53:7237–53. 10.1007/s12035-015-9582-7 26687231

[58] Bu L Baba H Yoshida N Miyake K Yasuda T Uchihara T. Biological Heterogeneity and Versatility of Cancer-Associated Fibroblasts in the Tumor Microenvironment. Oncogene (2019) 38(25):4887–901. 10.1038/s41388-019-0765-y 30816343

[59] Sahai E Astsaturov I Cukierman E DeNardo DG Egeblad M Evans RM. A Framework for Advancing Our Understanding of Cancer-Associated Fibroblasts. Nat Rev Cancer (2020) 20(3):174–86. 10.1038/s41568-019-0238-1 PMC7046529 31980749

[60] Dzobo K. Cancer-Associated Fibroblasts: Origins, Heterogeneity and Functions in Tumor Microenvironment. OMICS: A J Integr Biol (2020) 24:314–39. 10.1089/omi.2020.0023 32496970

[61] Paulsson J Ehnman M Östman A. PDGF Receptors in Tumor Biology: Prognostic and Predictive Potential. Futur Oncol (201) 10:1695–708. 10.2217/fon.14.83 25145436

[62] Hanley CJ Waise S Parker R Lopez MA Taylor J Kimbley LM. Spatially Discrete Signalling Niches Regulate Fibroblast Heterogeneity in Human Lung Cancer. bioRxiv (2020). 10.1101/2020.06.08.134270

[63] Zhou J Tang Z Gao S Li C Feng Y Zhou X. Tumor-Associated Macrophages: Recent Insights and Therapies. Front Oncol (2020) 10:188. 10.3389/fonc.2020.00188 32161718 PMC7052362

[64] Recalcati S Locati M Marini A Santambrogio P Zaninotto F De Pizzol M. Differential Regulation of Iron Homeostasis During Human Macrophage Polarized Activation. Eur J Immunol (2010) 40(3):824–35. 10.1002/eji.200939889 20039303

