# Supplementary figures and images for: The Ferroxidase Hephaestin in Lung Cancer: Pathological Significance and Prognostic Value
Source: Front Oncol. 2021 May 19;11:638856. doi: 10.3389/fonc.2021.638856 (PMC8170403; doi:10.3389/fonc.2021.638856)

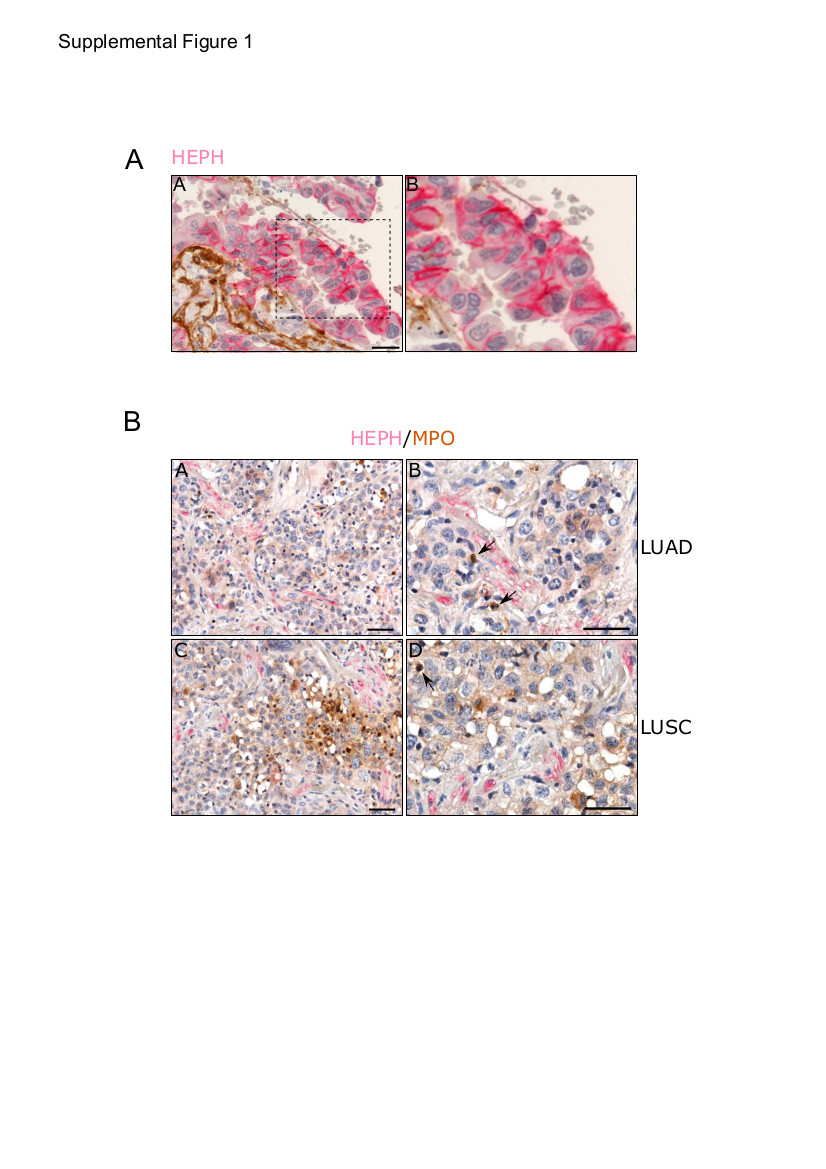

Supplement: Supplementary Figure 1 — (A) HEPH is clearly detected at the cell membrane is some neoplastic nests (panel A). A higher magnification of the dashed area is reported on panel B. (B) HEPH is not expressed by neutrophils. Representative microphotographs relative to lack of HEPH expression in neutrophils recognized by MPO immunoreactivity (indicated by arrows) in LUAD (A, B) and LUSC (C, D). Polymer detection system with AEC (red) chromogen for HEPH an d DAB (3,3′ -Diaminobenzidine) chromogen for CD14; scale bars, 50µm. [file Image_1.jpeg]

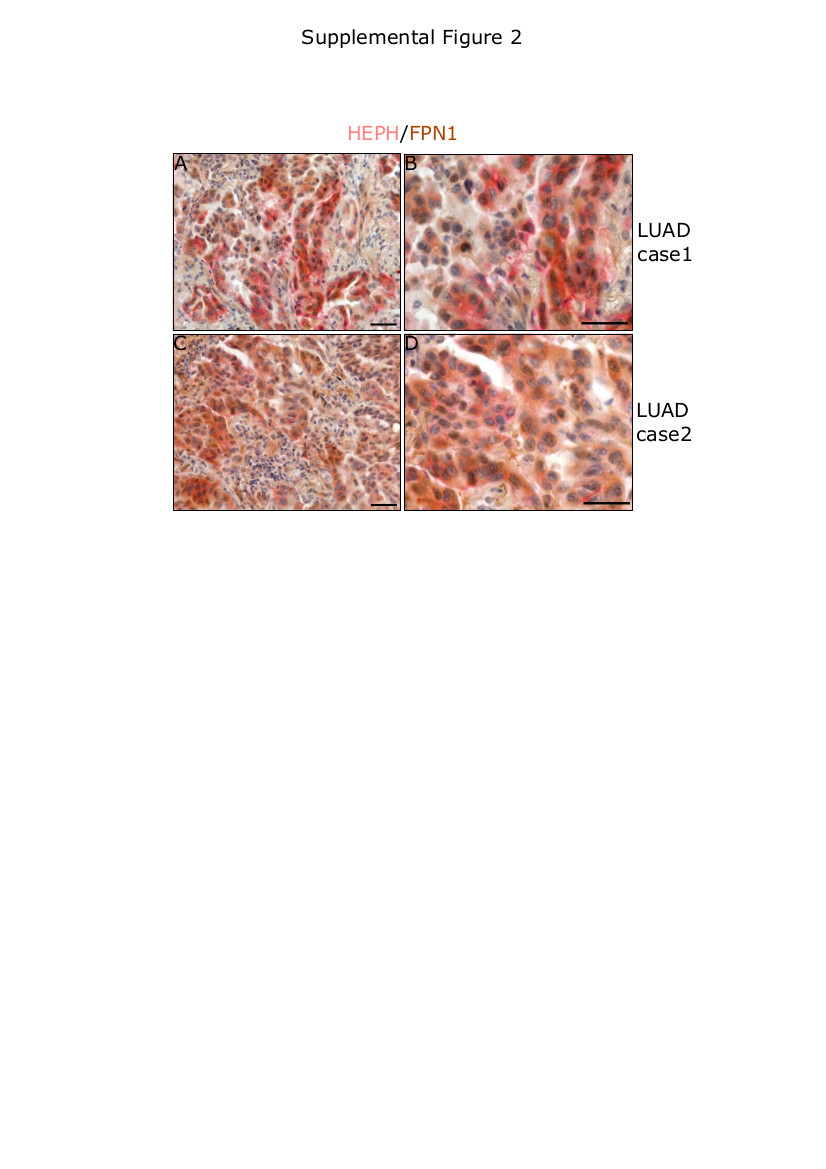

Supplement: Supplementary Figure 2 — HEPH co-expresses with FPN1 in some cancer nests. Representative microphotographs relative to HEPH/FPN1 co-expression in two cases of LUAD (A, B) and LUSC (C, D). Polymer detection system with AEC (red) chromogen for HEPH an d DAB (3,3′ -Diaminobenzidine) chromogen for CD14; scale bars, 50µm. [file Image_2.jpeg]

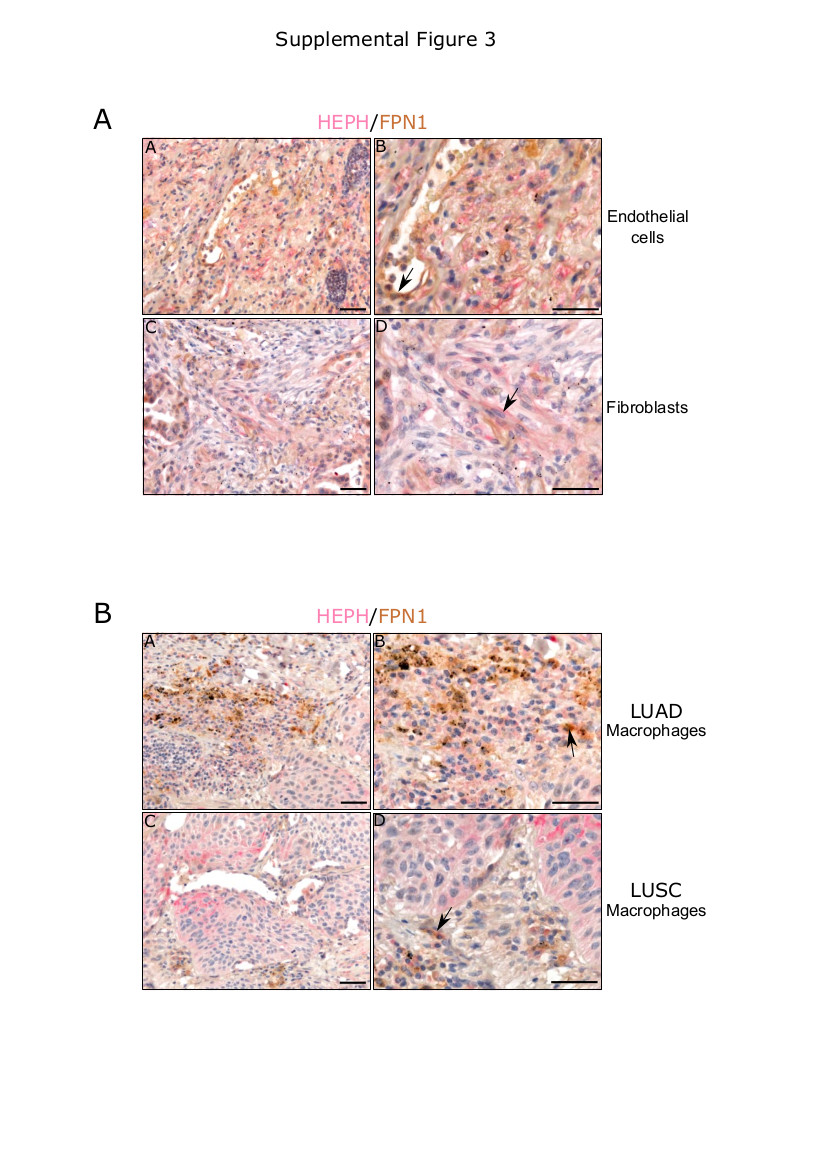

Supplement: Supplementary Figure 3 — HEPH/FPN1 are partially co-expressed on endothelial cells and stromal fibbroblasts, as well as on macrophages. (A) Representative microphotographs relative to HEPH/FPN1 co-expression on endothelial cells (A, B) and fibroblasts (C, D) (see black arrows). (B) Representative microphotographs relative to HEPH/FPN1 co-expression on macrophages in LUAD (A, B) and LUSC (C, D) (see black arrows). Polymer detection system with AEC (red) chromogen for HEPH an d DAB (3,3′ -Diaminobenzidine) chromogen for CD14; scale bars, 50µm. [file Image_3.jpeg]
